# Eye, Ear, Nose, and Throat

**Published:** 1895-02

**Authors:** 


### *SELECTIONS AND ABSTRACTS.*

---

### EYE, EAR, NOSE, AND THROAT.

Edited by Dunbar Roy, A.B., M.D.

### Trachoma.

In the October issue of the *Ophthalmic Record* will be found a translation by me of a recent article by Dr. Ed. Pergens "On the value of mechanical and surgical treatment of Chronic Trachoma," which was published in the *Klin Monättsh. für Augenheilkunde.* In this article Dr. Pergens publishes his experience with the various methods used in the treatment of this affection from the standpoint of a micro-anatomical examination. In other words he has undertaken to find by microscopical examination what procedures most completely destroy the trachomatous granules, and therefore which is the best. In his investigation he has taken pieces from the conjunctiva and tarsus of all the patients suffering with trachoma, has taken them from patients undergoing the different methods of treatment, and finally has taken them from patients before and after the treatment was instituted. He finds that all the surgical methods used are valuable, but more especially that method which is used by him, consisting, as it does, in not only scarifying the conjunctiva and emptying the trachoma follicles but even in cutting deep into the tarsus itself. He finds that none of the methods are complete cures in the true sense, but that they are certainly better than the old burning with copper and nitrate of silver.

In this connection it will be well to state the results of E. Venneman, showing the opinions of the French on the same subject. I quote from the *Annals of Ophthalmology and Otology:*

"Dr. Venneman has tried all the operative methods employed in treating granular lids, and they have *all* in his hands been productive of good results, and every one—expression of the granulations, excision of the cul-de-sac, scraping, curetting, the use of the hard brush on the everted mucosa, with and without previous scarification—has been equally successful."

With this experience he is inclined to believe, as Panas does, that every operation on the lids, attended by loss of blood, is of value in the treatment of granular ophthalmia. The hemorrhage acts by depletion of the local blood-vessels and lessens the activity of the inflammatory processes in acute and subacute forms of trachoma. In the chronic variety the benefit also springs from the destruction of the nutrient vessels.

Moreover in simple canthotomy and canthoplasty one cuts off a portion of the vascular supply to the lids, just as in excision of the superior cul-de-sac a great many arterioles furnished to the palpebral conjunctiva are divided. So it is in "grattage" curetting and the use of the hard brush there is indirect destruction of the conjunctival capillaries themselves. Partisans of one or the other of these various surgical methods of treating trachomatous lids appear to be working along the same lines. They desire, while removing or destroying the granular tissue, to leave intact the healthy conjunctiva. But Venneman believes, as the result of several years' study of the subject, that it is a great mistake to suppose it possible to be able to destroy the granulations without injury to the mucous membrane of which they form a part. Indeed the trachomatous granule is a transformed lymphatic corpuscle and is not a separate new formation, but is the conjunctiva itself equally hypertrophied and transformed into conjunctival embryonic tissue. The lymph corpuscle is a body absolutely innocent of all the damage done by the disease to the conjunctiva and by extension to the globe itself. The lymphatic follicle is merely an accidental element in trachoma. It may disappear without leaving any trace, and certainly never gives rise to cicatricial tissue. It is only in the large granulations, also, that one finds those bodies which have been called the corpuscles of Flemming. The number of normal lymph corpuscles in the diseased conjunctiva is very variable. The chief thing to be borne in mind is that whatever removes the embryonic tissue, constituting, with the trachoma bacillus, the disease called granular lids, kills off so much healthy conjunctiva. The author prefers a combined method of treatment and practices a superficial removal of the epithelium by Desmorré's scarifier, combined with a sublimate irrigation of 1:500 solution. On each of the following three days the conjunctiva is rubbed with tampons of cotton-wool saturated with the same solution. Cocaine is needed only for scarifying; there is usually no pain or reaction, but if any occurs cold compresses are to be applied. This it to be repeated daily if necessary. The results are most beneficial. After two or three days a false membrane forms over the conjunctiva; when rubbed off by the tampon does not bleed. The author also regards the appearance of the false membrane as a good sign and thinks that it indicates an early recovery from the disease. He ascribes the good results of this combined treatment to the direct action of the corrosive sublimate on the microbes and their morbid products. Relapses are less likely to occur if the patient's general health and surroundings receive attention.

H. Gefford, M.D. (*Medical Record*, August 25, 1894): This writer, differing somewhat from the two above, abstracts from whose writings have been given, has published his views in the above journal in an article entitled, "The Shady Side of the Surgical Treatment of Trachoma." The writer does not place in such high esteem the value of the various surgical methods of treating trachoma, as will be seen in the two abstracts above. He believes that the cases reported as cured have not been followed and such observations are entirely too short. His conclusions are as follows:

"These cases make it plain, I think, that surgical measures have not simplified the treatment of trachoma; on the contrary, they have made it altogether more imperative that he who uses them should be able to examine the eye with the greatest care and discrimination and should be prepared, if unusual emergencies arise, to treat them promptly and vigorously. Without these qualifications the physician had far better stick to the old bluestone and nitrate of silver treatment; for with these remedies the disease can always be improved or held in check and in many cases cured; whereas, if he uses surgical measures at all freely, while he may, as a rule, obtain more brilliant results, he is liable, at any time, to ruin an eye which otherwise would not be lost."

### THE TENOTOMY OF THE TENSOR TYMPANI IN CHRONIC MIDDLE EAR INFLAMMATION.

Hoffman reports the results in twenty-two cases operated upon in the Jena Clinic. Subjective sensations disappeared in four cases after the operation. In one case, in a year after the operation. The hearing power was improved in fifteen per cent.; unchanged in forty-five per cent.; diminished in forty per cent. For whispering speech, improvement in fifty per cent.; unchanged in thirty-five per cent.; diminished in fifteen per cent. The purulent discharge ceased in forty per cent.; in two cases there was no otorrhea at the time of the operation, and none developed since; in forty per cent. there has been a discharge. With regard to the hearing, improvement is to be expected only if there is no ankylosis of the stapes, no affection of the auditory nerve, and no extensive adhesions in the middle ear; a cure of the purulent discharge only in such cases as are not complicated by caries. The author considers that we have in tenotomy a conservative method of treatment, and that from the practice of this operation we have learned much releative to the clinical significance of perforations situated at the upper and lower extremities of the hammer respectively. In the former we may almost invariably expect caries, while the latter, on the contrary, are present in uncomplicated cases of purulent otitis media.

---

IN a most excellent article entitled "The Teeth of Our School-children: What Can be Done to Save Them," which was read in the Section on Dental and Oral Surgery at the last meeting of the American Medical Association, by Dr. J. C. M. McCoy, of Santa Ana, Cal., a condition of affairs exists among our school-children which is well worth serious consideration. The writer calls attention to the fact that ignorance and neglect are the causes of more diseases and loss of more teeth than all other causes combined. To show how the ordinary hygiene of the teeth is neglected, the writer had slips with the following questions distributed among the scholars in a school of five hundred pupils. The questions were all answered with the following results: Fifty cleaned their teeth twice daily; two hundred and seventy-five used a brush sometimes; while one hundred and seventy-five did not own a brush.

The writer further says that where there is so much neglect and so little real care of the mouth, it is not at all strange that the sixth year molars have to be sacrificed daily. It is, indeed, a question which should receive more attention in our homes and even in our schools.

